# The structure of depressive manifestations in preoperative cardiac surgery patients

**DOI:** 10.1192/j.eurpsy.2021.1832

**Published:** 2021-08-13

**Authors:** O. Nikolaeva, E. Nikolaev, N. Maksimova, E. Litvinova, A. Zakharova, G. Dulina

**Affiliations:** 1 Cardiosurgery, Republic Cardiology Clinic, Cheboksary, Russian Federation; 2 Medical Faculty, Ulianov Chuvash State University, Cheboksary, Russian Federation; 3 Social And Clinical Psychology, Ulianov Chuvash State University, Cheboksary, Russian Federation

**Keywords:** preoperative cardiac surgery patients, Depression, time perspective, psychological interventions

## Abstract

**Introduction:**

It is common knowledge that depressive disorders are prevalent in cardiac patients. The fact of a prospective heart surgery can have a negative effect on depressive manifestations in cardiac patients.

**Objectives:**

To describe representation and structure of depression in preoperative cardiac surgery patients and its correlation with the patients’ personal time perspective

**Methods:**

We used the Beck Depression Inventory to estimate the level and structure of depression in 60 cardiac surgery patients of both sexes and the Zimbardo Time Perspective Inventory to identify the patients’ personal time perspective.

**Results:**

We revealed depression of various manifestations in 53.4% of preoperative cardiac patients; 3.3% of them had severe depression, 11.7% – moderate depression, 8.3% – mild depression, 30.0% – minimal depression. The patients’ average level of depression was certainly higher than the standard one (t=3.295; р=.000). According to degree, the structure of depressive manifestations included asthenia, irritability, sleeping disorders, low sex drive, weight loss, pessimism, tearfulness, difficulty working, and difficulty taking decision. Two patients showed suicidal thoughts. We revealed a positive correlation between the depression level and a Negative-Past time perspective (r=.39) and a negative correlation with the Positive Past time perspective (r=-.27).

**Conclusions:**

We identified depressive manifestations in every second preoperative cardiac patient. Every sixth one has moderate or severe depression, which calls for special attention. Research in personal time perspective has good prospects for psychological interventions.

**Disclosure:**

No significant relationships.

